# Boussignac continuous positive airway pressure for the management of acute cardiogenic pulmonary edema: prospective study with a retrospective control group

**DOI:** 10.1186/1471-2261-7-40

**Published:** 2007-12-20

**Authors:** Willem Dieperink, Tiny Jaarsma, Iwan CC van der Horst, Wybe Nieuwland, Karin M Vermeulen, Hanka Rosman, Leon PHJ Aarts, Felix Zijlstra, Maarten WN Nijsten

**Affiliations:** 1 Thoraxcenter, Department of Cardiology. University Medical Center Groningen, University of Groningen, Hanzeplein 1 P.O. Box 30.001, 9700 RB Groningen, The Netherlands; 2 Surgical Intensive Care Unit. University Medical Center Groningen, University of Groningen, Hanzeplein 1 P.O. Box 30.001, 9700 RB Groningen, The Netherlands; 3 Department of Epidemiology. University Medical Center Groningen, University of Groningen, Hanzeplein 1 P.O. Box 30.001, 9700 RB Groningen, The Netherlands; 4 Department of Anaesthesiology. University Medical Center Groningen, University of Groningen, Hanzeplein 1 P.O. Box 30.001, 9700 RB Groningen, The Netherlands

## Abstract

**Background:**

Continuous positive airway pressure (CPAP) treatment for acute cardiogenic pulmonary edema can have important benefits in acute cardiac care. However, coronary care units are usually not equipped and their personnel not adequately trained for applying CPAP with mechanical ventilators. Therefore we investigated in the coronary care unit setting the feasibility and outcome of the simple Boussignac mask-CPAP (BCPAP) system that does not need a mechanical ventilator.

**Methods:**

BCPAP was introduced in a coronary care unit where staff had no CPAP experience. All consecutive patients transported to our hospital with acute cardiogenic pulmonary edema, a respiratory rate > 25 breaths/min and a peripheral arterial oxygen saturation of < 95% while receiving oxygen, were included in a prospective BCPAP group that was compared with a historical control group that received conventional treatment with oxygen alone.

**Results:**

During the 2-year prospective BCPAP study period 108 patients were admitted with acute cardiogenic pulmonary edema. Eighty-four of these patients (78%) were treated at the coronary care unit of which 66 (61%) were treated with BCPAP. During the control period 66 patients were admitted over a 1-year period of whom 31 (47%) needed respiratory support in the intensive care unit. BCPAP treatment was associated with a reduced hospital length of stay and fewer transfers to the intensive care unit for intubation and mechanical ventilation. Overall estimated savings of approximately € 3,800 per patient were achieved with the BCPAP strategy compared to conventional treatment.

**Conclusion:**

At the coronary care unit, BCPAP was feasible, medically effective, and cost-effective in the treatment of acute cardiogenic pulmonary edema. Endpoints included mortality, coronary care unit and hospital length of stay, need of ventilatory support, and cost (savings).

## Background

Many cases of acute cardiogenic pulmonary edema (ACPE) are treated with an oxygen mask and pharmacologic treatment including diuretics and vasodilators [1,2]. When such treatment is not sufficient, additional ventilatory support may be useful [3,4]. Traditionally this has been achieved via endotracheal intubation and mechanical ventilation, an approach that usually requires intensive care unit (ICU) admission. Continuous positive airway pressure (CPAP) applied by a face mask is a feasible alternative. Several randomized clinical trials [5-8] report a decreased rate of intubation and decreased hospital length of stay for patients assigned to mask-CPAP compared with conventional therapy with oxygen alone. These trials were performed either at the emergency department or at the ICU and not in an environment such as a coronary care unit (CCU) which is often not suited to use mechanical ventilators that are normally necessary for CPAP.

A simple CPAP device which does not require a ventilator might therefore be useful to treat patients with ACPE in the CCU and reduce the need for more invasive treatment in the ICU. The Boussignac CPAP (BCPAP) system is a small lightweight plastic cylinder that is directly connected to a face mask which has been shown to be effective for ACPE in the emergency department and in prehospital care [9,10]. Although no data are available on the effectiveness of BCPAP in the CCU we found no convincing medical arguments why BCPAP could not be effective in the CCU. We hypothesized that the major obstacle in treating patients with BCPAP in the CCU would be organizational. We studied if implementation of BCPAP in the CCU was practically feasible, medically effective and cost reducing. For this purpose all patients admitted to our hospital with ACPE were studied.

## Methods

### Boussignac CPAP system

The BCPAP face mask system (Vygon, 95440 Ecouen, France) which is CE-approved and approved by the Food and Drug Administration is a simple and lightweight (10 g) disposable cylindrical plastic device without sensors, mechanical valves and without heavy tubing. A jet flow of air and or oxygen generated in this plastic tube creates a flow dependent pressure [9-14]. We used an air/oxygen blender (Bird 3800™ Microblender, Palm Springs, USA) for full control of the inspired oxygen fraction (FiO_2_). BCPAP can generate a fraction of FiO_2 _that approaches 100% [11]. The generated pressure is proportional to the flow applied: 3 cm H_2_O at a flow of 8 L/min, 5 cm H_2_O at a flow of 15 L/min and 10 cm H_2_O at a flow of 23 L/min. This was measured during CPAP treatment with a handheld electronic pressure analyzer (Testo 505-P1, Almere, the Netherlands).

### Patients

This study was conducted in a regional university hospital with a 12 bed CCU and 4 ICU's with 47 beds. ACPE was defined as acute dyspnea associated with a past of ischemic heart disease or cardiomyopathy and physical signs consistent with pulmonary edema including a respiratory rate > 25 breaths/min and a peripheral arterial oxygen saturation (SpO_2_) < 95% while receiving oxygen [3].

We studied all adult patients admitted to our hospital with respiratory failure due to ACPE. Exclusion criteria were known evidence of severe pulmonary pathology, Glasgow coma scale < 9 and in the prospective group the inability to wear a face mask (facial deviations, non-cooperative patient). The first ten patients treated with BCPAP on the CCU were observed by the medical and nursing staff of the ICU to ensure a safe and sufficient CPAP treatment.

The institutional review board of the hospital approved the study design that compared a historical control group with a prospective BCPAP group. Verbal consent was obtained and recorded from patients to use their medical data. (METc 2004.195)

### BCPAP group and control group

**Figure 1:**
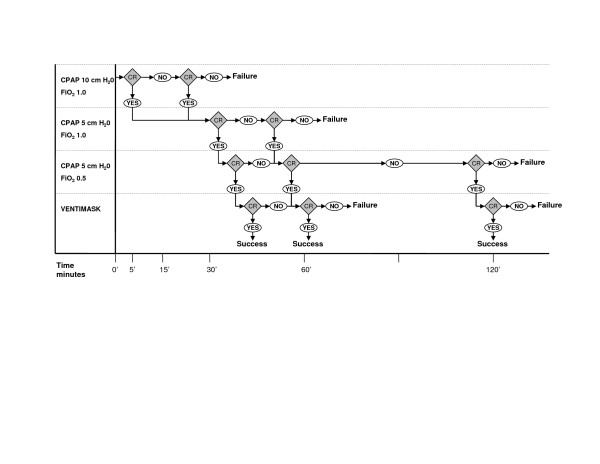
**Algorithm for BCPAP treatment in patients with ACPE in the CCU**. Protocol as used for patients treated with BCPAP in the CCU. Since ACPE should respond within 30 minutes to BCPAP therapy, this protocol required early and frequent assessments of the patients' response. From left to right the duration of BCPAP treatment is displayed and from top to bottom the decreasing level of respiratory support. CR: clinical response is defined as an improvement in respiration rate and SpO_2 _to at least > 95%. Success was defined as achievement of CR with breathing oxygen through a ventimask. Note that, depending on the patients' response the protocol leads to a minimal duration of BCPAP of 30 minutes and a maximal duration of 2 hours.

Patients in the intervention group received BCPAP with an initial pressure of 10 cm H_2_O and an FiO_2 _of 80–100%. BCPAP pressure and FiO_2 _were decreased when there was a clinical improvement in SpO_2 _and heart rate. Patients were preferably kept in a sitting position during BCPAP. In order to facilitate successful implementation of BCPAP both medical and nursing staff was involved in the implementation process. During a training session of 45 minutes we introduced the BCPAP system as well as a protocol (Figure 1) for applying CPAP. The training session included theory and practical use of CPAP and was distributed to all staff by an experienced ICU nurse according a standardized teaching package to ensure consistency. CCU staff was instructed to use BCPAP not for other indications than ACPE.

Data from the prospective BCPAP cohort of patients were compared with the data from a historical control group. The prospective part of the study was conducted over a period of 24 months. Patients in the control group were admitted in the same CCU and hospital in a previous 12 month period and treated with oxygen through non-rebreathing mask (FiO_2 _approximately 100%), ventimask (FiO_2 _40–60%) or nasal catheter (3 L/min; FiO_2 _30%) and on ICU admission with endotracheal intubation. For this purpose we evaluated all records from patients admitted to the CCU and the ICU's for ACPE.

Data on pharmacological treatment with diuretics, opioids and nitroglycerine as well as patient acceptance, complications, SpO_2_, heart rate, CCU length of stay, hospital length of stay, hospital mortality and long term mortality were collected.

An economic evaluation was carried out to estimate costs of the strategy that included BCPAP therapy compared with conventional treatment. This evaluation was performed from a hospital perspective, and all relevant costs made between admission and discharge from the hospital were taken into account. Where possible, costs were valued according to Dutch standard prices [15]; if no standard prices were available, costs were estimated based on true resources used and time invested by the hospital personnel. Cost categories included were: stay in CCU (€ 1,112/day), stay in ICU (€ 1,733/day), admission to hospital ward (€ 490/day), BCPAP therapy (€ 42), personnel cost BCPAP therapy (€ 23) and endotracheal intubation (€ 377). Based on costs of both conventional treatment and BCPAP, and the percentages of patients admitted to the CCU and ICU, total costs for both strategies were calculated and compared.

### Statistical analysis

Data are presented as medians and associated interquartile ranges (IQR) or means with standard deviation (SD) for continuous variables, or as group percentages for categorical variables. Statistical analysis was performed using Student's t, Mann Whitney, Wilcoxon signed rank test, Fisher exact or Chi-square test. Overall comparisons were based on intention-to-treat: all patients treated at the CCU or ICU for ACPE were compared between the control and the BCPAP period, irrespective of whether they actually received BCPAP. All statistical analyses were performed using commercially available software (SPSS 14.0, SPSS, Chicago, Illinois, USA).

## Results

**Figure 2:**
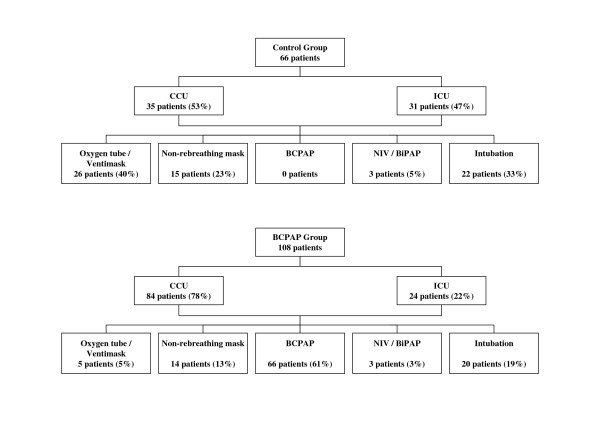
**Study design**. First all patients with ACPE were examined at the emergency department by a cardiologist and depending on his findings transferred to CCU or ICU. The control group consisted of all patients admitted to our hospital with ACPE over a one year period. The BCPAP group covered all patients with ACPE over a two year period. Note the considerable shift of ICU admissions to CCU admissions in the BCPAP group (p < 0.001). The number of patients treated with BCPAP in the control group was zero since this option was then not available.

Sixty-six patients were included in the control group and 108 patients in the BCPAP group (Figure 2). Patients in both study groups were predominantly male and had a mean age of 72 years. Table 1 shows the characteristics of both groups on an intention-to-treat basis. Pharmacological treatment did not differ between the groups. Eighty-four (78%) of the patients in the BCPAP group were admitted to the CCU, of which 66 were actually treated with BCPAP.

**Table 1:** Patient characteristics

	**Control**	**BCPAP**	**p-value**
N	66	108	
Duration of the study	1 year	2 years	
Age in yrs, mean, ± SD	72 ± 11	72 ± 11	-
Male, n (%)	46 (70)	69 (64)	-
Medication, n (%)			
Loop-diuretics	56 (85)	97 (90)	-
Opioids	34 (51)	47 (44)	-
Nitroglycerine	29 (44)	39 (36)	-
Admission CCU, n (%)	35 (53)	84 (78)	< 0.01
Admission ICU, n (%)	31 (47)	24 (22)	< 0.01
CCU stay in days, median (IQR)	3 (2–5)	2 (2–3)	-
CCU stay in days, mean (SD)	2.0 (2.3)	2.3 (2.7)	-
ICU stay in days, median (IQR)	2 (2–5)	4 (2–7)	< 0.01
ICU stay in days, mean (SD)	2.0 (3.5)	1.3 (3.4)	-
Hospital stay in days, median (IQR)	16 (8–24)	8 (2–16)	< 0.01
Hospital stay in days, mean (SD)	20.2 (23.5)	13.9 (17.9)	0.047
Hospital survivors, n of total (%)	50 (76)	84 (78)	-
6 month survival, n of total (%)	49 (74)	81 (75)	-

Comparison of the two patients groups studied with intention-to-treat analysis. The median CCU and ICU stay is only given for those patients who were admitted to the CCU and ICU respectively. In contrast, the mean CCU and ICU stay are calculated for the whole group. Comparisons were performed with the Fisher exact test, Chi-square test, Student-t test and the Mann Whitney test. IQR = Interquartile range.

No problems in the practical use of the BCPAP system were identified. Eighteen patients who were admitted to the CCU were eligible for BCPAP but did not receive this treatment and received oxygen alone. The main reason was because there were insufficient signs of serious respiratory distress and staff believed that the patients (baseline SpO_2 _91%. IQR 88–93) showed adequate spontaneous improvement. The median duration of BCPAP treatment was 105 minutes (IQR 60–172 minutes). Pressure measurements showed a predicted CPAP level in 76% of the BCPAP patients. In only two patients a flow of more than 23 L/min had to be given to achieve the desired BCPAP level. Sixty-four of 66 patients maintained stable gas exchange after BCPAP was stopped. Two of the 66 patients required follow-up respiratory support in the ICU after BCPAP treatment in the CCU. One patient with a large myocardial infarction and support with an intra-aortic balloon pump had to be intubated and the other received ventilator mask-CPAP. No other complications of the BCPAP treatment were reported and 88% of the staff assessed the implementation of BCPAP as simple and uncomplicated.

### Saturation and heart rate

Baseline median SpO_2 _was lower in the BCPAP group than in the control group (88% versus 91%, p < 0.01). Both groups showed a significant increase in SpO_2 _after 2 hours (Table 2). Nevertheless the increase of SpO_2 _was higher in the BCPAP group compared to the control group (+8% versus +3%, p < 0.01) with a similar SpO_2 _after 2 hours. Heart rate in the BCPAP group was significantly decreased after 2 hours of treatment (median 104/min to 86/min), in contrast to the unchanged heart rate in the control group (median 102). In the 2 to 6 hours after discontinuation of BCPAP, the saturation or heart rate did not deteriorate again.

**Table 2:** Physiological responses before and after BCPAP in subgroup of CCU patients.

	**Control**	**BCPAP**	**p-value**
N	35	84	
Heart rate baseline	102 (83–125)	104 (86–119)	-
Heart rate after 120 min	102 (82–117)	86 (76–99)*	0.03
Mean arterial pressure baseline	101 (85–118)	103 (79–123)	-
SpO_2 _baseline	91 (88–94)	88 (82–92)	< 0.01
SpO_2 _after 120 min	94 (92–95)*	95 (92–97)*	-

Data are median (IQR), SpO_2 _= peripheral arterial oxygen saturation. The Wilcoxon signed rank test was used to test differences between data collected at baseline and after 120 minutes. * Significant (p < 0.05) within group improvement at 120 minutes compared to baseline.

### Intubations, length of stay and outcome

After implementing BCPAP on the CCU, the number of intubations showed an absolute decrease of 14% (p < 0.001) compared to the control period with conventional treatment. Likewise the number of ICU admissions showed an absolute decrease of 25% (p < 0.001) compared to the control period.

The median CCU-stay in de BCPAP group was 1 day with a median hospital length of stay of 6 days. In the control group the median CCU-stay was 2 days and the median hospital length of stay was 14 days. There was no significant difference between the mortality rates in both groups (BCPAP 22% versus control 24%).

### Costs

As a shift was found in route of admittance, resulting in more patients in the BCPAP group admitted to the CCU compared to the ICU, costs changed accordingly. In the control group, mean total costs for ACPE patients were € 13,754 per patient.

In the BCPAP group (where 61% of the patients actually received BCPAP) mean total costs were € 9,968. This resulted in a saving of € 3,787 per candidate for the new treatment strategy compared to the traditional strategy.

## Discussion

We demonstrated that for patients with acute cardiogenic pulmonary edema it is possible to use a simple form of mask-CPAP. The introduction of BCPAP at our CCU decreased endotracheal intubations and the related ICU admissions. Our study also underscores that CPAP, as delivered by mask, is superior to conventional oxygen treatment in improving oxygenation and decreasing heart rate. Moreover, no complications were observed with the use of the BCPAP system on our CCU. The decreasing number of endotracheal intubations with related ICU admissions after implementation of BCPAP on our CCU led to notable cost reductions. Savings were approximately € 3,800 per candidate for the strategy that included BCPAP compared to the conventional strategy.

The treatment of ACPE with non-invasive CPAP was already described in de first half of the previous century by both Poulton [16] and Barach [17]. Nowadays there is extensive evidence that supports the use of non-invasive CPAP [7,1]. CPAP delivered by mask was shown to be beneficial in several randomized clinical trials [5-8] as stated in the introduction. However, these studies were not performed at a CCU or a likewise environment. In general CPAP together with pharmacological therapy achieves rapid clinical improvement but heart failure – of which ACPE is a cardinal manifestation – is still associated with high in-hospital mortality and a poor long-term prognosis [18], as also is seen in our patients (Table 1).

ACPE patients show significant improvement in the PaO_2_/FiO_2 _ratio, subjective dyspnea score, and respiratory and heart rates with CPAP [19,20]. At the same time CPAP can reduce venous return, decrease ventricular filling pressures and improve cardiac performance. In our patients, CPAP produced a rapid physiological and symptomatic improvement in patients with ACPE, especially within the first hours, as also been observed with other CPAP modalities [21]. More complicated forms of non-invasive ventilation, as bilevel ventilation, showed no additional benefits in the treatment of ACPE compared to CPAP [7,22]. Therefore, clinicians must be aware that if a rapid physiological and symptomatic improvement does not occur, the etiology of respiratory insufficiency might not be acute cardiogenic pulmonary edema. In fact our protocol allowed no more than 120 minutes of BCPAP treatment because other causes for respiratory distress may then be present and intubation might be more appropriate in this setting.

A tight fitted facemask can cause discomfort but it is fundamental for achieving adequate positive pressure in the airways during CPAP. The patients at our CCU were systematically queried about their experiences with BCPAP treatment and they found that the reduction of dyspnea outweighed any discomfort of the facemask. These findings were exemplified by a patient who was successfully treated during many episodes of ACPE with BCPAP: although initially wary about a facemask he subsequently easily accepted it for many times [23]. Although most nurses in our CCU had limited expertise and experience in the field of mask-CPAP they could apply BCPAP effectively after a short instruction period.

The effectiveness of BCPAP has also been demonstrated in prehospital settings [9]. Future studies focusing on the early administration of BCPAP in patients with ACPE – in a pre-hospital setting such as an ambulance – would add insight into the clinical advantages and cost benefits of early CPAP treatment of patients with ACPE.

There are some limitations of the present study. First, the data from the control group is retrospective and ACPE is not always easy to diagnose. Based on the evaluation of all patient records great care was taken to include all patients who were admitted to our hospital with ACPE according to established criteria [3]. Second, in the prospective BCPAP group the treatment of patients might have differed slightly due to ongoing advances in treatment of patients with ACPE. For comparison, treatment of patients with ST-elevation myocardial infarction improved because of more widespread and rapid use of percutaneous coronary intervention. But in the treatment of ACPE no major or minor changes in the treatment have occurred in our institution. Finally, calculated savings were based on assumptions that are related with the reimbursement system in the Netherlands and therefore should be interpreted with caution when transposed to other institutions.

## Conclusion

The results of this study underscore the utility of the Boussignac CPAP system as a simple, effective and cost saving modality on a CCU for patients with ACPE.

## Competing interests

The author(s) declare that they have no competing interests.

## Authors' contributions

WD, HR, and MN executed the study, collected data and drafted the manuscript. WD, TJ, IH, WN, KV, FZ and MN participated in conception, design, analysis and interpretation. TJ, IH, WN, LA, FZ and MN assisted in writing the manuscript. TJ, LA, FZ, and MN revised the manuscript critically for important intellectual content, and all gave final approval of the manuscript.

## Pre-publication history

The pre-publication history for this paper can be accessed here:


